# Clinicopathologic Features of Patients with Non-Small Cell Lung Cancer Harboring the EML4-ALK Fusion Gene: A Meta-Analysis

**DOI:** 10.1371/journal.pone.0110617

**Published:** 2014-10-31

**Authors:** Ying Wang, Shumin Wang, Shiguang Xu, Jiaqi Qu, Bo Liu

**Affiliations:** 1 Department of Pathology, Shenyang Medical College, Shenyang, Liaoning, People's Republic of China; 2 Thoracic Department, General Hospital of Shenyang Military Area Command, Shenyang, Liaoning, People's Republic of China; H. Lee Moffitt Cancer Center & Research Institute, United States of America

## Abstract

**Background:**

The frequencies of EML4-ALK fusion gene in non-small cell lung cancer (NSCLC) with different clinicopathologic features described by previous studies are inconsistent. The key demographic and pathologic features associated with EML4-ALK fusion gene have not been definitively established. This meta-analysis was conducted to compare the frequency of the EML4-ALK fusion gene in patients with different clinicopathologic features and to identify an enriched population of patients with NSCLC harboring EML4-ALK fusion gene.

**Methods:**

The Pubmed and Embase databases for all studies on EML4-ALK fusion gene in NSCLC patients were searched up to July 2014. A criteria list and exclusion criteria were established to screen the studies. The frequency of the EML4-ALK fusion gene and the clinicopathologic features, including smoking status, pathologic type, gender, and EGFR status were abstracted.

**Results:**

Seventeen articles consisting of 4511 NSCLC cases were included in this meta-analysis. A significant lower EML4-ALK fusion gene positive rate was associated with smokers (pooled OR = 0.40, 95% CI = 0.30–0.54, *P*<0.00001). A significantly higher EML4-ALK fusion gene positivity rate was associated with adenocarcinomas (pooled OR = 2.53, 95% CI = 1.66–3.86, *P*<0.0001) and female (pooled OR = 0.61, 95% CI = 0.41–0.90, *P* = 0.01). We found that a significantly lower EML4-ALK fusion gene positivity rate was associated with EGFR mutation (pooled OR = 0.07, 95% CI = 0.03–0.19, *P*<0.00001). No publication bias was observed in any meta-analysis (all *P* value of Egger's test >0.05); however, because of the small sample size, no results were in the meta-analysis regarding EGFR gene status.

**Conclusion:**

This meta-analysis revealed that the EML4-ALK fusion gene is highly correlated with a never/light smoking history, female and the pathologic type of adenocarcinoma, and is largely mutually exclusive of EGFR.

## Introduction

Since the early 2000s, a more thorough understanding of the molecular biology of non-small cell lung cancer (NSCLC) has led to major advances in treatment of this neoplasm. Distinct subtypes of NSCLC are driven by specific genetic alterations and are sensitive to the inhibitors of the activated oncogenic pathways [1]. Identification of mutations in the epidermal growth factor receptor (EGFR), K-ras gene, and most recently the echinoderm microtubule-associated protein-like 4 and anaplastic lymphoma kinase (EML4-ALK) fusion gene have had a decisive impact on the treatment of NSCLC.

The EML4-ALK fusion gene was first described in 2007 by Soda and colleagues [2], who screened a cDNA library derived from the cancer tissue of a 62-year-old Japanese male patient with NSCLC. Inversions within the short arm of chromosome 2 (involving 2p21 and 2p23; approximately 12 Mb apart) results in the formation of this fusion gene, which leads to constitutive ALK kinase activation, possessing potent oncogenic activity both *in vitro* and *in vivo* [2]–[4]. In these tumors, the EML4-ALK fusion gene is the determinant of critical growth pathways, resulting in the activation of PI3K/AKT and MAPK/ERK pathways downstream [1]. A tyrosine kinase inhibitor, crizotinib, has been shown to selectively inhibit growth of cancer cells with EML4-ALK fusion gene.

According to previous studies, the presence of this fusion gene is more likely present in patients with specific demographic characteristics. Some of previous investigators have identified EML4-ALK predominantly in young female non-smokers with adenocarcinoma [2], [5], [6], whereas other reports have identified this fusion gene in different populations [7]–[9]. The clinicopathologic features associated with EML4-ALK have not been completely established. The small overall number of patients enrolled in each study may explain these discrepancies. In order to identify an enriched population of patients with NSCLC harboring EML4-ALK fusion gene and to identify more useful information on candidate selection for ALK tyrosine kinase inhibitor therapy, we performed this meta-analysis to compare the frequency of the EML4-ALK fusion gene in patients with different clinicopathologic features.

## Materials and Methods

The meta-analysis was performed, according to the PRISMA statement (Preferred reporting items for systematic reviews and meta-analyses) [10], including search strategy, selection criteria, data abstraction and data analysis.

### 1 Search Strategy

We searched the Pubmed and Embase databases for all articles on the association between the EML4-ALK fusion gene and NSCLC up to July 2014. The medical subject headings and key words used for search were “EML4-ALK fusion gene, human”, ”EML4-ALK”, “nonsmall cell lung cancer”, “carcinoma, non-small-cell lung”, “non small cell lung carcinoma”, “non-small-cell lung carcinoma”, “non-small cell lung cancer” and “non-small cell lung carcinoma”. Related articles were also searched to broaden the search. All citations and abstracts acquired were reviewed. The references of the articles acquired were also searched by hand. No language restrictions were imposed.

### 2 Inclusion and Exclusion Criteria

Eligible studies had to meet the following criteria: (1) the association between the EML4-ALK gene and the clinicopathologic features of patients with NSCLC was explored; (2) the diagnosis of NSCLC was made according to the pathologic results; (3) studies with full text articles; and (4) sufficient data for estimating an odds ratio (OR) with a 95% confidence interval (CI).

The exclusion criteria were: (1) duplicate data; (2) abstract, comment, review, and editorial; and (3) insufficient data were reported.

### 3 Data Abstraction

The following items were collected: first name of first author; year of publication; country of origin; ethnicity of patients; number of enrolled patients; frequency of the EML4-ALK fusion gene; detection method; and the clinicopathologic features (gender, smoking status, pathologic type, and EGFR status). All the above informations were carefully independently extracted from all eligible publications by two investigators. If the two investigators could not reach a consensus, the result was reviewed by a third investigator.

### 4 Statistical Analysis

The strength of the association between the EML4-ALK fusion gene and the clinical features of NSCLC was estimated by odds ratios (ORs) and 95% confidence intervals (CIs). The chi-square based Q test was used to assess the statistical heterogeneity among studies. When heterogeneity existed, a random-effects model based on the DerSimonian and Laird method was used to calculate the pooled OR of each study; otherwise, a fixed-effects model based on the Mantel-Haenszel method was used. Publication bias was examined using funnel plot and Egger's test. All analyses were performed using the Review Manager program (RevMan version 5.3) and the statistical software package R (version 2.15.1) [11]. All tests were two-sided and the significance level was set at 0.05.

## Results

**Figure 1:**
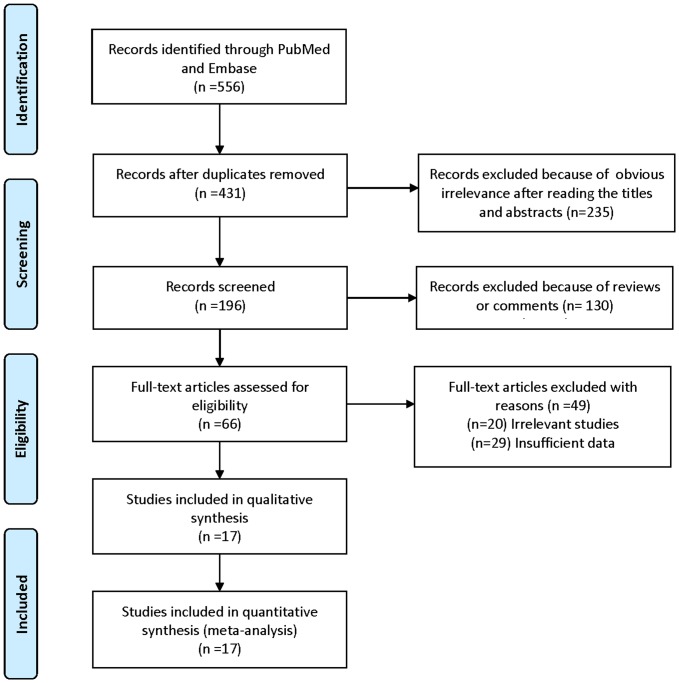
Flow diagram of study selection in this meta-analysis.

The acquisition process of studies is depicted in Fig. 1. A total of 17 articles [5]–[8], [12]–[24] met our inclusion criteria and were included in the final meta-analysis, which consisted of 4511 NSCLC cases. The characteristics of each study are presented in Table S1. Of the 17 studies, 13 involved Asians, 2 involved Caucasians, and 2 involved a mixed population. The protein detection methods included RT-PCR (10 studies), FISH and IHC (2 studies), FISH (3 study), and IHC (2 study).

**Figure 2:**
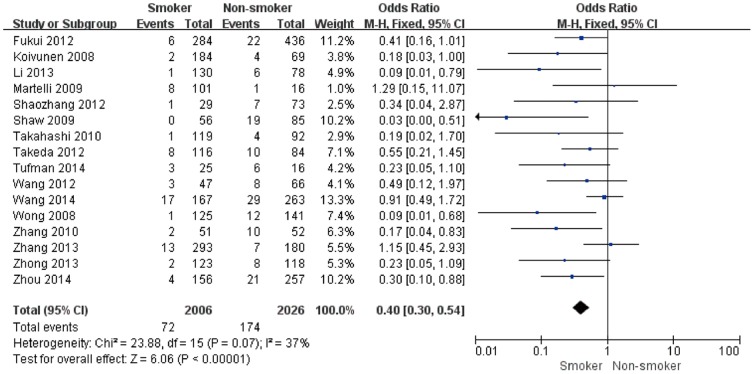
Forest plots of studies with an association between the EML4-ALK fusion gene and smoking status.

There were 16 studies which had been performed to detect the EML4-ALK fusion gene in smokers and non-smokers with NSCLC. The pooled frequency of EML4-ALK was 3.59% (72/2006) and 8.59% (174/2026) in smokers and non/light smokers, respectively. Overall, when all the eligible studies were pooled into the meta-analysis (Fig. 2), no significant heterogeneity was observed (I^2^ = 37%, *P* = 0.07), thus we chose the fixed-effects model and found that a significantly lower EML4-ALK fusion gene positivity rate was associated with smokers (pooled OR = 0.40, 95% CI = 0.30–0.54, *P*<0.00001).

**Figure 3:**
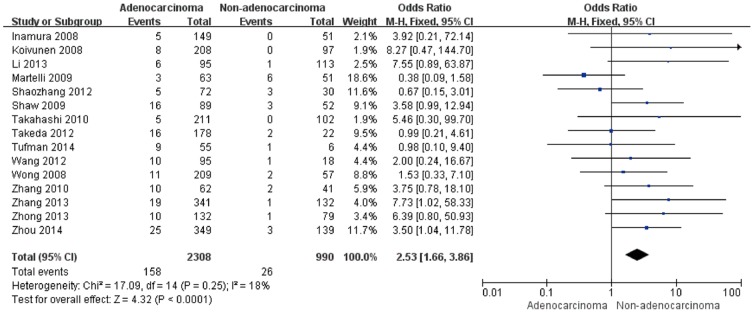
Forest plots of studies with an association between the EML4-ALK fusion gene and pathologic type.

We identified 15 studies that addressed the frequency of the EML4-ALK fusion gene in adenocarcinomas and non-adenocarcinomas. The pooled frequency of EML4-ALK was 6.85% (158/2308) and 2.63% (26/990) in adenocarcinomas and non-adenocarcinomas, respectively. When all of the eligible studies were pooled into the meta-analysis (Fig.3), no significant heterogeneity was observed (I^2^ = 18%, *P* = 0.25), thus we chose the fixed-effects model and found that a significantly higher EML4-ALK fusion gene positivity rate was associated with adenocarcinomas (pooled OR = 2.53, 95% CI = 1.66–3.86, *P*<0.0001).

**Figure 4:**
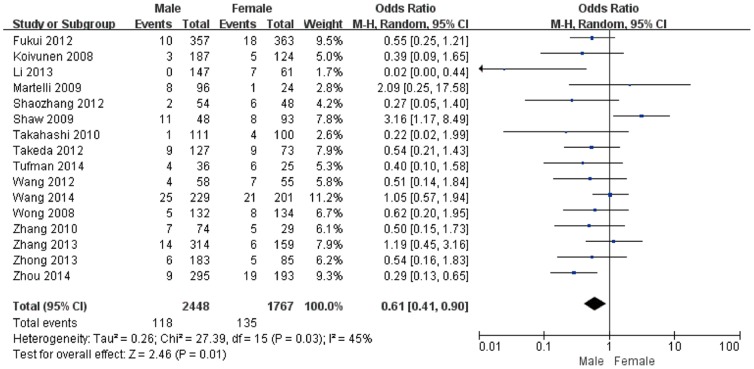
Forest plots of studies with an association between the EML4-ALK fusion gene and gender.

Gender information was available for 16 studies, and included a total of 2265 male and 1682 female patients. EML4-ALK was detected in 118 (4.82%) males and 135 (7.64%) females. The results of this meta-analysis are shown in Fig. 4. Overall, when all of the eligible studies were pooled into the meta-analysis, significant heterogeneity was observed (I^2^ = 45%, *P* = 0.03), thus the random-effects model was chosen. EML4-ALK mutant tumors were more likely to be women (pooled OR = 0.61, 95% CI = 0.41–0.90, *P* = 0.01).

**Figure 5:**
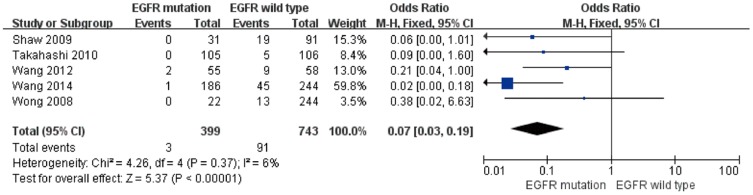
Forest plots of studies with an association between the EML4-ALK fusion gene and EGFR.

Four studies presented clinical data for an association between the EML4-ALK and EGFR genes. The results of the meta-analysis are shown in Fig. 5. In 399 NSCLC patients with a EGFR mutation, there were 3 (0.75%) patients harboring EML4-ALK. In 743 NSCLC patients with wild-type EGFR, there were 91 (12.25%) patients harboring the EML4-ALK gene. All of the eligible studies were pooled into the meta-analysis, and no significant heterogeneity was observed (I^2^ = 6%, *P* = 0.37); thus, the fixed-effects model was chosen. We found that a significantly lower EML4-ALK fusion gene positivity rate was associated with EGFR mutant tumors (pooled OR = 0.07, 95% CI = 0.03–0.19, *P*<0.00001).

No publication bias was noted in any meta-analysis (all *P*-values of Egger's tests were >0.05) as shown in Table S2. However, because of the small sample size, there were no results in the meta-analysis regarding the association of EML4-ALK and EGFR status.

## Discussion

Based on the studies included in this meta-analysis, the incidence of EML4-ALK ranged from 1.6% to 16.4% in patients with NSCLC [5]–[8], [12]–[24]. Although the frequency of EML4-ALK in NSCLC patients was low, because the incidence of NSCLC is increasing worldwide, the absolute number of NSCLC patients harboring the EML4-ALK fusion gene is noteworthy. Indeed, clinicopathologic features could help us to precisely select an enriched population with this specific molecular subset of NSCLC in clinical practice. The present meta-analysis of 17 studies, which included 4511 cases, revealed that the EML4-ALK fusion gene is highly correlated with a never/light smoking history, female and the pathologic type of adenocarcinoma, and is largely mutually exclusive of EGFR.

The frequencies of EML4-ALK in NSCLC patients with different smoking statuses described in previous reports are inconsistent. In the first report of this fusion gene in NSCLC [2], EML4-ALK was detected in five patients, two of whom were noted to have a smoking history. In the subsequent studies prior to 2009, EML4-ALK was variably detected in smokers and non-smokers [18], [19], [25], [26], which suggested a lack of association between the presence of EML4-ALK and smoking history. In 2009, Shaw and colleagues [7] reported that the presence of EML4-ALK is more likely in patients with a never/light smoking status. In the current meta-analysis, we found that EML4-ALK is strongly associated with a never/light smoking history.

Previous studies have reported that EML4-ALK occurs at a significantly higher frequency in adenocarcinoma [5]–[7], [12], [15], [18], [19], while some studies have also reported that EML4-ALK occurs at a similar frequency in non-adenocarcinomas [8], [13], [14], [16], [17]. Our meta-analysis confirmed that EML4-ALK is significantly associated with adenocarcinoma. Compared with non-adenocarcinomas, the overall OR of EML4-ALK in lung adenocarcinomas was 2.53.

The reported frequencies of EML4-ALK between males and females have been inconsistent. Shaw et al. [7] demonstrated that EML4-ALK patients are more likely to be males. In contrast, EML4–ALK fusion genes were observed predominantly in females in other studies [15], [18]. A meta-analysis performed by Li et al. [6] found that there was no significant difference between the male and female groups. However, the current meta-analysis showed that patients with EML4-ALK positive tumors are more likely to be female.

There may be potential interactions between the gender and smoking status. In Asian countries, the smoking rate in women was much lower than man, and lower than women in western countries [27]–[30]. This meta-analysis involved more studies performed in Asian population, and more female patients were non-smokers, which may be the reason of the inconformity of gender between the meta-analysis by Li [6] and this meta-analysis.

It has been reported that the concurrence of ALK fusion and EGFR mutations is a rare event (3/399) [17]–[19], [23], [26]. In patients with an EGFR mutation, the presence of EML4-ALK was significantly lower than patients with wild-type EGFR. Zhang et al. [5] reported that the presence of ALK fusions is correlated with wild-type EGFR status. He identified 1 of 12 patients who had the EML4-ALK fusion gene and an EGFR mutation; the patient was a Chinese female non-smoker with a histologic adenocarcinoma [5]. Although patients with EML4-ALK share several clinicopathological features with patients with EGFR mutations, such as never/light smoking history and adenocarcinoma histology, the current meta-analysis found that EML4-ALK fusion gene was mutually exclusive for EGFR mutations, thus suggesting a distinct genetic subtype of lung adenocarcinoma.

In previous studies, EML4-ALK was more frequently found in young patients [7], [8], [13]; however, in this meta-analysis, the age data was not pooled due to the different points of demarcation.

Crizotinib is a small molecule tyrosine kinase inhibitor which was originally developed as an inhibitor of mesenchymal–epithelial transition growth factor (c-MET). In 2010, a single-arm phase 1 trial involving crizotinib in patients with NSCLC harboring EML4-ALK was completed [31]. The overall response rate was 57%and the disease control rates were 87% at 8 weeks and 66% at 16 weeks [31]. The findings of the current meta-analysis may facilitate patient selection for EML4-ALK inhibitor therapy.

Some limitations of this meta-analysis should be acknowledged. First of all, there was no randomized controlled trial (RCT) involved in this meta-analysis. Given that there was no data from RCTs for this topic, we limited our scope to these prospective and retrospective trials to obtain the best data available. Secondly, due to the small proportion of EML4-ALK-positive NSCLC patients, the sample size available for analysis was small. Thirdly, previous studies were mainly performed in east-Asia. There were only two studies involved Caucasians, and two involved a mixed population. Whether the different race contributes to the difference of EML4-ALK remains unknown. The results of this meta-analysis may be biased by race. Finally, we could not analyze the association between EML4-ALK and patient age because of the different sets of data presentation in age.

In summary, our meta-analysis demonstrated that the incidence of the EML4-ALK fusion gene was significantly higher in never or light smokers, women, patients with EGFR wild type and adenocarcinomas. However, the studies enrolled in this meta-analysis all had a small sample size; thus, further studies with large sample sizes are needed to confirm our findings.

## Supporting Information

Table S1 **A descriptive summary of the studies used in the meta-analysis.**
(DOCX)

Table S2 **The results of Egger's tests for publication bias.**
(DOCX)

Checklist S1 **PRISMA checklist.**
(DOC)
