# Supplementary material for: Clinicopathologic Features of Patients with Non-Small Cell Lung Cancer Harboring the EML4-ALK Fusion Gene: A Meta-Analysis
Source: PLoS One. 2014 Oct 31;9(10):e110617. doi: 10.1371/journal.pone.0110617 (PMC4215846; doi:10.1371/journal.pone.0110617)
Supplement: Table S1 — A descriptive summary of the studies used in the meta-analysis. (DOCX) [file pone.0110617.s001.docx]

| Study | Year | Source of Patient | Ehnicity | No. | Frequency | Method |
| --- | --- | --- | --- | --- | --- | --- |
| Fukui | 2012 | Japan | Asian | 720 | 3.9%(28/720) | RT-PCR,IHC |
| Inamura | 2008 | Japan | Asian | 200 | 2.5%(5/200) | RT-PCR |
| Koivunen | 2008 | U.S. and Korea | Caucasian (138) and Asian (167) | 305 | 3%(8/305) | RT-PCR |
| Li | 2013 | China | Asian | 208 | 3.4%(7/208) | RT-PCR |
| Martelli | 2009 | Italy | Caucasian | 120 | 7.5%(9/120) | RT-PCR |
| Shaozhang | 2012 | China | Asian | 102 | 8%(8/102) | RT-PCR |
| Shaw | 2009 | U.S. | Asian (9) and Non-Asian (132) | 141 | 13%(19/141) | Fish,IHC |
| Takahashi | 2010 | Japan | Asian | 313 | 1.6%(6/313) | RT-PCR |
| Takeda | 2012 | Japan | Asian | 200 | 9.0%(18/200) | RT-PCR |
| Tufman | 2014 | Germany | Caucasian | 61 | 16.4%(10/61) | FISH |
| Wang | 2012 | China | Asian | 113 | 9.7%(11/113) | FISH |
| Wang | 2014 | China | Asian | 430 | 10.7%(46/430) | FISH |
| Wong | 2008 | Hong Kong | Asian | 266 | 4.9%(13/266) | IHC |
| Zhang | 2010 | China | Asian | 103 | 11.6%(12/103) | RT-PCR |
| Zhang | 2013 | China | Asian | 473 | 4.2%(20/473) | Fish,IHC |
| Zhong | 2013 | China | Asian | 268 | 4.1%(11/268) | RT-PCR |
| Zhou | 2014 | China | Asian | 488 | 5.73%(28/488) | IHC |
