# Supplementary material for: Clinicopathologic Features of Patients with Non-Small Cell Lung Cancer Harboring the EML4-ALK Fusion Gene: A Meta-Analysis
Source: PLoS One. 2014 Oct 31;9(10):e110617. doi: 10.1371/journal.pone.0110617 (PMC4215846; doi:10.1371/journal.pone.0110617)
Supplement: Table S2 — The results of Egger's tests for publication bias. (DOCX) [file pone.0110617.s002.docx]

| Meta-analysis | Egger's test | |
| --- | --- | --- |
|  | *t* value | *P* value |
| Smoking | -1.36 | 0.197 |
| Pathology | -1.07 | 0.311 |
| Gender | 0.03 | 0.973 |
| EGFR | – | – |
